# Hypophysitis induced by ipilimumab and nivolumab combination therapy for advanced renal cell carcinoma: A case report

**DOI:** 10.1016/j.eucr.2021.101661

**Published:** 2021-03-26

**Authors:** Aoi Motonaga, Shotaro Nakanishi, Kei Tanaka, Sho Nishida, Keiichiro Izumi, Seiichi Saito

**Affiliations:** Department of Urology, Graduate School of Medicine, University of the Ryukyus, Okinawa, 903-0215, Japan

**Keywords:** Hypophysitis, Ipilimumab-Nivolumab, Renal cell carcinoma, Headache, Ipi/Nivo, ipilimumab and nivolumab, RCC, renal cell carcinoma, irAE, immune-related adverse event, CT, computed tomography, MRI, magnetic resonance imaging

## Abstract

Ipilimumab and nivolumab combination therapy is effective against unresectable or metastatic renal cell carcinoma. However, it is associated with many immune-related adverse events, including hypophysitis that is difficult to diagnose early because of non-specific initial symptoms. Herein, we report the case of a 54-year-old man with metastatic renal cell carcinoma who developed hypophysitis after receiving ipilimumab and nivolumab combination therapy. The initial symptom was headache. However, endocrine tests showed decreased levels of cortisol, free thyroxine and thyroid-stimulating hormone. Moreover, magnetic resonance imaging revealed pituitary enlargement. Accordingly, we diagnosed hypophysitis and immediately started hydrocortisone replacement therapy, which improved the symptoms.

## Highlights

• Ipilimumab/nivolumab therapy is effective against unresectable or metastatic RCC.
• Hypophysitis is an immune-related adverse event with non-specific initial symptoms.
• Its delayed diagnosis may cause substantial mortality due to adrenal insufficiency.
• Therefore, suspicious symptoms should be monitored and further examined.
• This minimizes immune-related adverse events due to immune checkpoint inhibitors.

## Introduction

Ipilimumab and nivolumab (Ipi/Nivo) combination therapy has been proven effective against unresectable or metastatic renal cell carcinoma (RCC). However, it is associated with many immune-related adverse events (irAEs). One such irAE is hypophysitis. The overall incidence of hypophysitis induced by Ipi/Nivo combination therapy in RCC was reported to be 4.0%, and the proportion of serious cases, such as those with Common Terminology Criteria for Adverse Events grade 3 or 4, was 2.7%.^1^

The initial symptoms of hypophysitis are non-specific and could be overlooked; however, these can lead to substantial mortality due to adrenal insufficiency.^2^ Therefore, prompt diagnosis and timely initiation of appropriate treatment are important.

To our knowledge, no study has been reported about hypophysitis induced by Ipi/Nivo combination therapy for RCC.

Herein, we report a case of hypophysitis induced by Ipi/Nivo combination therapy and diagnosed by the onset of slight headache, wherein prompt treatment intervention and good control of hypophysitis could be achieved.

## Case presentation

**Fig. 1:**
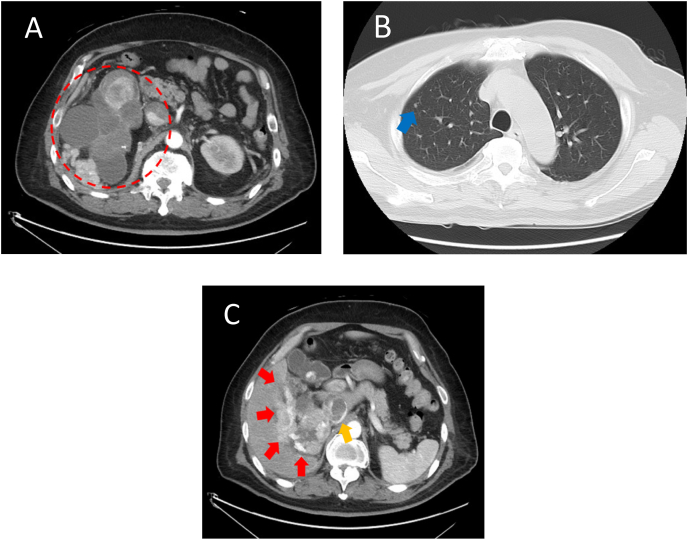
Computed tomography reveals a hypervascular tumor accompanied by a cystic lesion with a maximum diameter of 16 cm in the right kidney (A), with lung metastasis (B) and invasion into the inferior vena cava, liver, and adrenal gland (C).

A 54-year-old man visited a local doctor with a chief complaint of exertional breathlessness and fever. His blood test results showed a decreased hemoglobin level of 7.6 g/dl. Computed tomography (CT) revealed a hypervascular tumor accompanied by a cystic lesion with a maximum diameter of 16 cm in the right kidney, with lung metastasis and invasion into the inferior vena cava, liver, and adrenal gland (Fig. 1). These findings led to a diagnosis of right RCC (cT4N0M1), and he was referred to our hospital for treatment. He was categorized as having an International Metastatic RCC Database Consortium Risk Score of 3 on the basis of anemia, Karnofsky performance status <80%, and a period of less than 1 year from the time of diagnosis to the start of treatment. Consequently, we initiated Ipi/Nivo combination therapy as the initial treatment.

**Fig. 2:**
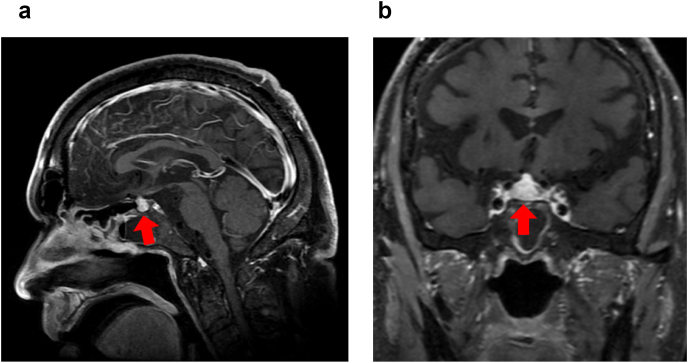
Magnetic resonance imaging reveals pituitary enlargement.

CT performed after the second course of the therapy revealed that the tumor in the right kidney showed stable disease and that the lung metastasis showed progressive disease. Simultaneously, the patient complained of headache. A blood biochemical analysis revealed a low sodium (121 mmol/l), high potassium (5.4 mmol/l), and low glucose (65 mg/dl) levels. A hematological analysis revealed an increased eosinophil fraction of 10% of leukocytes. Based on these findings, we suspected hyposecretion of cortisol and performed endocrine tests. Although his cortisol level was low (1.59 μg/dl), adrenocorticotropic hormone level was normal (25.3 pg/ml). In addition, both free thyroxine (0.80 ng/dl) and thyroid stimulating hormone (0.18 μIU/ml) levels were low. Subsequently, magnetic resonance imaging (MRI) was performed which revealed pituitary enlargement (Fig. 2). Based on these findings, we diagnosed the patient with hypophysitis as an irAE. Accordingly, we discontinued the administration of Ipi/Nivo combination therapy and started oral hydrocortisone replacement therapy (15 mg/day). His headache resolved immediately, and laboratory data showed improvement in hyponatremia, hyperkalemia, and hypoglycemia. Four days after initiating the oral administration of hydrocortisone, we resumed single-agent nivolumab therapy. Since then, he has had no recurrence of hypophysitis and has been able to continue nivolumab therapy.

## Discussion

In various cancers, the incidence of hypophysitis induced by treatment using the anti-cytotoxic T lymphocyte antigen-4 antibody ipilimumab and the anti-programmed cell death-protein 1 antibody nivolumab has been reported to be 5.6% and 0.5%, respectively. However, the incidence of hypophysitis induced by Ipi/Nivo combination therapy is 8.8%, and this combination therapy has a tendency to show a higher incidence and grade of irAEs than does single-agent monotherapy.^3^

The most common signs and symptoms of hypophysitis are nausea, vomiting, weakness, fatigue, hypotension, fever, and headache. Visual disturbance is less frequent because pituitary swelling is not usually large enough to affect the optic chiasma.^4^ These initial symptoms are non-specific and similar to those accompanying cancer progression. Therefore, differentiating and diagnosing this condition is sometimes difficult. Delayed diagnosis and treatment of hypophysitis can lead to severe outcomes like adrenal crisis in some cases.^2^

A diagnosis is typically made on the basis of hormonal and radiological evaluations of the pituitary gland. When hypophysitis is suspected, both pituitary hormones and target tissue hormones should be evaluated. The main MRI finding is pituitary enlargement or nodularity.^4^ However, some patients show a normal pituitary gland on MRI.^3^

In the present case, the initial symptom was headache, which was non-specific and likely to be overlooked. However, we carefully examined and diagnosed hypophysitis because the headache appeared after the administration of immune checkpoint inhibitors, which seemed to induced the headache.

The treatment plan for hypophysitis is based on whether it is symptomatic. In patients with symptoms such as headache, visual disturbance, or hypotension, immune checkpoint inhibitors should be discontinued and hormone replacement therapy should be initiated. If both adrenal and thyroid functions are impaired, cortisol replacement therapy should be administered before levothyroxine to avoid the worsening of adrenal function. When the clinical symptoms improve, treatment with immune checkpoint inhibitors can be restarted.^5^

In this case, we could immediately initiate hormone replacement therapy on the basis of the early diagnosis and could prevent severe complications.

## Conclusion

This is the first case of hypophysitis induced by Ipi/Nivo combination therapy for advanced RCC.

Diagnosing hypophysitis as an irAE is difficult because of its non-specific clinical symptoms. However, a delayed diagnosis can be critical for the patient. Therefore, clinicians should look out for suspicious symptoms such as headache, and perform detailed examination to minimize the damage caused by irAEs. In addition, early treatment of hypophysitis can shorten the interval of immune checkpoint inhibitor therapy, which in turn affects cancer progression.

## Consent

Written informed consent was obtained from the patient for publication of this case report.

## Declaration of competing interestCOI

None.

## Author contributions

Aoi Motonaga, Shotaro Nakanishi, Kei Tanaka, Sho Nishida, Keiichiro Izumi: conceptualization, investigation, writing-original draft, writing-review and editing. Seiichi Saito: Supervision.

## Funding

This research did not receive any specific grant from funding agencies in the public, commercial, or not-for-profit sectors.
